# Assess the Variability and Robustness of an Aluminum-Based Adsorption–Precipitation Method for Virus Detection in Wastewater Samples

**DOI:** 10.3390/microorganisms13061186

**Published:** 2025-05-23

**Authors:** Lorena Casado-Martín, Marta Hernández, José M. Eiros, Antonio Valero, David Rodríguez-Lázaro

**Affiliations:** 1 Microbiology Area, University of Burgos, Plaza Misael Bañuelos s/n, 09001 Burgos, Spain; lcasado@ubu.es; 2 Centre for Emerging Pathogens and Global Health, University of Burgos, Plaza Misael Bañuelos s/n, 09001 Burgos, Spain; 3 Microbiology Area, Faculty of Medicine, University of Valladolid, 47002 Valladolid, Spain; marta.hernandez.perez@uva.es (M.H.); jmeiros@uva.es (J.M.E.); 4 Department of Food Science and Technology, UIC Zoonosis y Enfermedades Emergentes (ENZOEM), CeiA3, Universidad de Córdoba, Campus Rabanales, 14014 Córdoba, Spain; bt2vadia@uco.es

**Keywords:** wastewater, epidemiology, virus surveillance, one health, quality control, aluminum-based adsorption–precipitation

## Abstract

Wastewater-based molecular epidemiology enables the surveillance of both symptomatic and asymptomatic individuals in a non-invasive, cost-effective, rapid, and early-detection manner. The use of wastewater analysis to monitor the prevalence of viral pathogens in a given population has increased significantly since the COVID-19 pandemic. These studies typically involve three main steps: viral concentration, nucleic acid extraction, and DNA/RNA quantification. However, the absence of a standardized methodology remains a major limitation, hindering result comparability across studies. Among the available viral concentration techniques, aluminum-based adsorption–precipitation is one of the most commonly used due to its simplicity, efficiency, and low cost. This study evaluates the robustness and variability of the viral concentration and nucleic acid extraction steps by implementing different process controls in wastewater samples across 122 independent experiments. Additionally, correlations between viral recovery efficiencies and relevant physicochemical parameters were also analyzed (*n* = 600). The results indicate that, despite the overall robustness of the method, the concentration step exhibits the highest variability (CV = 53.82%), which accounted for 53.73% of the overall variability. In addition, our results show that, on average, 0.65 logarithmic units were lost during the viral concentration step. Furthermore, viral recovery rates were influenced by seasonality and sample characteristics, while no significant correlation was observed with pH or conductivity. These findings highlight the importance of process controls, confirming the robustness of the methodology, and identifying key parameters that should be considered in future studies for improved data interpretation.

## 1. Introduction

Wastewater-based epidemiology (WBE) has emerged as a powerful tool for monitoring public health threats at the population level. Specifically, in the early 20th century, further advances in pathogen detection via wastewater occurred. During the 1920s, the microorganism responsible for typhoid fever was isolated from wastewater samples [1], emphasizing the role of this matrix in the spread of infectious diseases. By 1939, WBE was employed to monitor poliovirus [2]. However, it was in 2003 when the World Health Organization (WHO) formally recommended the use of WBE for tracking this pathogen in populations [3]. This environmental epidemiology has expanded beyond the detection of pathogens, being used for monitoring other molecules such as pharmaceuticals compounds or drugs consumption, as a mirror of the community health status [4,5]. Moreover, biomarkers, such as histamines related to allergies, prostaglandins reflecting oxidative damage, and endocrine disruptors found in everyday products, are also tracked using this methodology [6].

However, the surveillance of zoonotic agents in wastewater samples had received only limited attention until recent years [7]; for example waste water samples were used for monitoring isolated outbreaks of Salmonella [8] or norovirus and hepatitis A virus [9]. However, the COVID-19 pandemic increased the interest in this type of epidemiology; This approach has been adopted by national, regional, and local governments, as well as universities, research institutions, and private companies [10] to track the prevalence of SARS-CoV-2. By 2023, WBE was being applied in over 3000 locations across more than 70 countries and regions [7].

Like any methodology, WBE has its own advantages and disadvantages. One of the most significant challenges is the lack of standardized, universal protocols particularly for virus concentration and nucleic acid extraction. Various methodologies exist for each step of the process, complicating the harmonization of results and making cross-study comparisons difficult [11]. As viruses are often present at low concentrations in aquatic environments, concentrating viral particles from large volumes of water prior to molecular detection or quantification is necessary and adds complexity [12]. A wide range of concentration methods have been developed. These include electropositive and electronegative filtration [13,14], ultrafiltration [15], polyethylene glycol (PEG) precipitation [16], aluminum-based adsorption–precipitation [17], ultracentrifugation [18], skimmed-milk flocculation [19], VIRADEL or ViroCap (Scientific Methods, Granger, IN, USA), filtration [18,20,21], ionic exchange [22], or nanoceram filters (Argonide, Sanford, FL, USA).

The aluminum-based adsorption–precipitation method is the third most used, after protocols based on ultrafiltration and PEG precipitation [23]. Briefly, this method consists of forming positive charge flocs, by adding aluminum trichloride to water or wastewater samples, where negatively charged viruses, at neutral pH, were adsorbed. Then, flocs are separated by centrifugation and concentrated viral particles are released and resuspended with an elution buffer, often a beef extract solution. This viral concentration methodology is characterized by its simplicity, adaptability, efficiency, and low cost which allows t concentration of both enveloped and non-enveloped viruses [24]. While its practical advantages are well established, few studies have rigorously evaluated its robustness, variability, and sensitivity to environmental factors under real-world conditions.

Due to the different types of methodologies, with high variable recovery efficiencies, further studies need to be conducted to better characterize each one [25]. Furthermore, environmental samples such as sewage are highly variable between different scenarios (e.g., geographical areas or seasons). Consequently, for the same concentration method different recovery rates are obtained in a long-term analysis of a given area. Environmental samples contain a lot of molecules which can affect the process at the three main methodology phases: virus concentration, nucleic acid extraction, and PCR amplification. To handle this issue, seeding with an appropriate surrogate virus for each sample is recommended as a whole process control to obtain information on the virus recovery [26,27,28]. Surrogate should be a virus morphologically and genetically similar to the target and not present naturally in the tested water. Murine norovirus and mengovirus are two of the already established process controls [29].

This study aims to fill this gap by systematically assessing the robustness and variability of the aluminum-based adsorption–precipitation method for viral detection in wastewater. Over 600 samples collected from twelve locations over multiple seasons are used to examine the influence of key factors such as physicochemical parameters (pH, conductivity), sampling location, and environmental conditions on viral recovery. Process controls were applied at critical stages of the method to evaluate each step independently, focusing on viral concentration and nucleic acid extraction. To our knowledge, this is the first large-scale, long-term study to critically evaluate the performance of this widely used viral concentration method under real-world conditions. By identifying the main sources of variability and establishing performance baselines, this work contributes to the ongoing efforts to standardize methodologies in WBE and improve the reliability of virus surveillance in environmental samples.

## 2. Materials and Methods

### 2.1. Viruses

Mengovirus (MgV) vMC0 (CECT 100000), was used in this study. MgV is a non-enveloped virus from the Picornaviridae family and has been recognized as a process control by ISO 15216-1, 2017 [30]. This ensures that viral concentration and extraction procedures align with international standards for method validation. Two different MgV titer lots were used in this study (Lot 1 and Lot 2) with 2.11 × 10^6^ and 2.49 × 10^7^ infective units/mL, and 28 and 34 independent experiments were performed for each one, respectively.

### 2.2. Virus Detection Strategy

**Figure 1:**
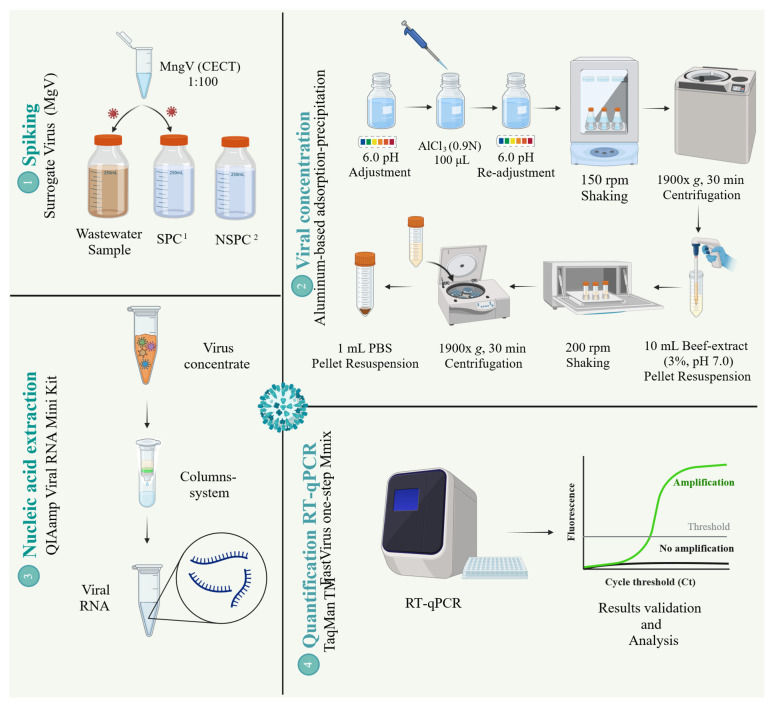
Workflow scheme. ^1^ SPC: Sample Process Control. ^2^ NSPC: Negative Sample Process Control. Created in BioRender (BioRender.com). Casado, L. (2025).

Figure 1 shows the workflow followed in this study.

#### 2.2.1. Process Controls Used in This Study

Three process controls were included in each experiment a Sample Process Control (SPC), a Negative Process Control (NSPC), and an Extraction Control (EC) [12,27] (Figure 1). The SPC consisted of 200 mL of autoclaved tap water—the same analyzed volume for samples–which were inoculated at the beginning of the analysis with 100 µL of a 1:100 dilution of the original Mengovirus stock. The SPC simulated an ideal recovery process, ensuring the accuracy of the entire method. A negative process control (NSPC) was included in all analyses to detect potential cross-contamination during processing. This control was processed identically to the SPC but without the MgV spiking. Additionally, an extraction control (EC) was included by inoculating 3 mL of the sterilized tap water—the approximate final volume obtained after virus concentration method—with the same amount of MgV, simulating a perfect concentration method. This control was used to evaluate the efficiency of the nucleic acid extraction step and how this step influenced the whole process. This control involved only the extraction and RT-qPCR quantification steps.

#### 2.2.2. Virus Concentration Step

The aluminum-based adsorption–precipitation method followed was a slight modification of the protocol described in [17]. A total of 200 mL of either pre-inoculated or non-inoculated samples were disposed to 250 mL PPCO centrifuge bottles (Thermo Fisher Scientific, Rochester, WA, USA). Then, pH was adjusted to 6.0 using 1 M HCl. Then, Al(OH)_3_, formed by adding 1 part 0.9N AlCl3 solution (Acros organics, Geel, Belgium) to 100 parts of sample, allows the virus precipitation. The pH was then readjusted to 6.0 using 10 M NaOH, followed by mixing in an orbital shaker (Luckham R300, Cole-Parmer, Vernon Hills, IL, USA) at 150 rpm for 15 min. The precipitated viruses were collected by centrifugation at 1900× *g* for 30 min (Centrikon T-124, Kontron Instruments, Linz, Austria Spain). The resulting pellets were resuspended in 10 mL of 3% beef extract solution at pH 7.0 (Lab-Lemco powder, Oxoid, Madrid, Spain) and transferred to 50 mL centrifuge tubes (CORNING CentriStar, Corning, NY, USA). Then, samples were shaken for 10 min at 200 rpm and then centrifuged again at 1900× *g* for 30 min (GR 20 22, Jouan, Thermo Fisher Scientific, Waltham, MA, USA). Finally, the pellet was resuspended in a 1 mL of phosphate buffered saline (PBS, SIGMA, Merk Life Science, Madrid, Spain). The whole method was carried out at room temperature. The final volume was approximately 3 mL. Samples were stored at −80 °C for less than 24 h before further processing.

#### 2.2.3. Nucleic Acid Extraction Step

Nucleic acid extraction was performed using the QIAamp Viral RNA Mini Kit (Qiagen, Hilden, Germany), following the manufacturer’s instructions [31,32]. A 150 µL aliquot of the previously described mixture was used, resulting in 60 µL of eluted RNA.

#### 2.2.4. Virus Quantification by RT-qPCR

The control virus (MgV) was quantified using One Step RT-qPCR in a 10 µL reaction volume containing 2.5 µL of extracted RNA. All reactions used the TaqMan Fast Virus 1-Step Master Mix (Applied Biosystems, Waltham, MA, USA) and were carried out by duplicate. The primers and probes sequences, along with their final concentrations were as specified in ISO 15216-1:2007 (ISO, 2017). Thermal cycling conditions were adapted to the TaqMan Fast Virus 1-Step Master Mix protocol and were as follows: reverse transcription (RT) at 50 °C for 15 min, an initial denaturation at 95 °C for 2 min, and 45 cycles of amplification at 95 °C for 15 s and 60 °C for 1 min. To assess potential RT-qPCR inhibitors, undiluted and ten-fold diluted MgV RNA were tested in duplicate. RT-qPCRs were performed in a QuantStudio 5 thermocycler (Applied Biosystems, US), and results were analyzed using the QuantStudio Design & Analysis desktop software (v1.5.1) (Applied Biosystems, US). To standardize comparative analyses, a fixed threshold was set manually. While the software automatically set the threshold at 10 times the standard deviation of the fluorescence baseline, this was manually adjusted to 0.07 to enhance practical consistency.

### 2.3. Methodology Robustness Study

The heterogeneity of the results was analyzed for different steps in the process. First, for the whole analytical method using SPC results. Second, for the nucleic acid extraction and amplification based on EC results. And finally, for the aluminum-based adsorption–precipitation procedure studying the differences between the average of the two replicates for each experiment for SPC and EC. Assuming that, RT-qPCR variability is the same for both SPC and EC because it was carried out at the same time and conditions.

### 2.4. Recovery Rates

The recovery rates of the 600 samples (Supplementary Material Table S1) were calculated as follows:

(1) $$recovery\;\left(\%\right)={100\times 2}^{Ct\;SPC-Ct\;Sample}$$

where Ct SPC represents the mean cycle threshold—set at 0.07—for the two SPC values obtained, and Ct Sample corresponds to the mean cycle threshold for each individual sample and experiment; both run in duplicate in each RT-qPCR and experiment.

### 2.5. Physicochemical Data

Physicochemical data, pH, and conductivity for each wastewater sample in each sampling date were measured during the collection process using the Hanna HI98194 Instrument (Hanna Instruments, Eibar, Spain). Data were collected for a total of 600 samples, from twelve different areas during fifty different dates.

### 2.6. Statistical Analysis and Plots

All statistical analyses were performed using R (version 4.4.0, 2024-04-24, ucrt). Normality was assessed using the Shapiro test function, while homogeneity of variance was evaluated with Levene’s test from the car package within *dplyr*. The relationship between recovery rates and sample groups was analyzed using a non-parametric Kruskal–Wallis test, given the non-normal distribution of data and the independence of groups. Post hoc comparisons were conducted using the *dunn.test* function from the *dunn test* package, applying the Benjamini–Hochberg correction for multiple comparisons.

Bland–Altman plots were generated using the *BlandAltmanLeh* package. The average of the differences and the 95% intervals of confidence were calculated as ± 1.96 *sd. Scatter plots and boxplots were created with *ggplot2*, while violin plots were generated using the *vioplot* package. Linear regression lines in scatter plots were fitted using the *lm* function.

## 3. Results

### 3.1. Robustness of the Methodology

**Figure 2:**
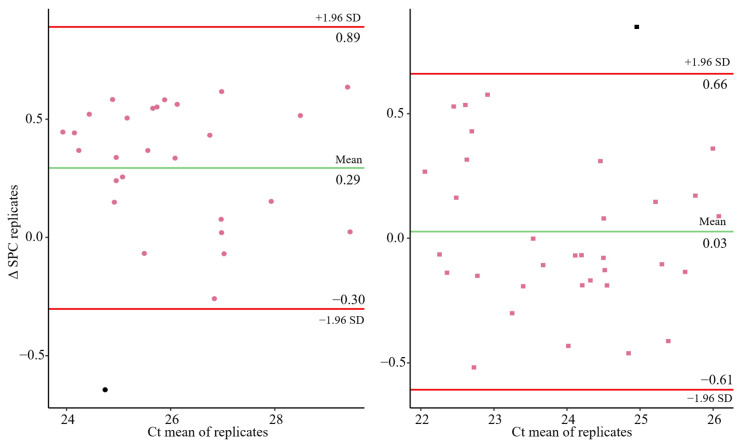
Bland–Altman plot for the differences between the two Sample Process Control (SPC) replicates performed in each independent experiment for Lot1 (**left**) and Lot 2 (**right**). Green line represents the mean of the results. Red lines represent the 95% confidence interval of the results. Black dots represent outliers.

**Figure 3:**
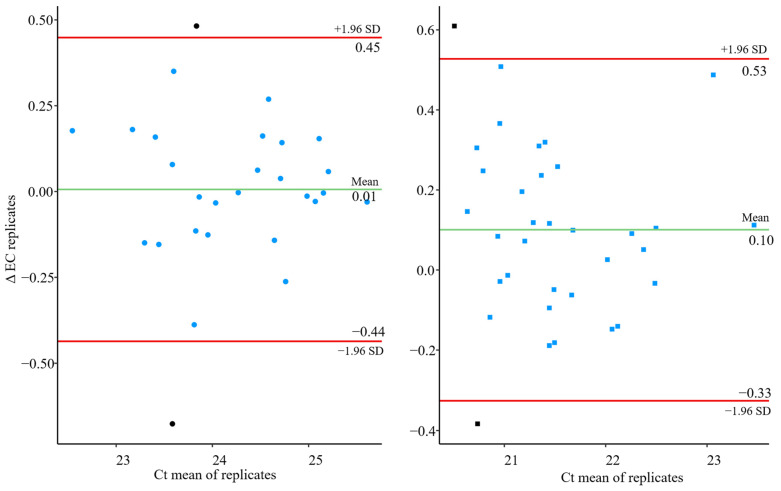
Bland–Altman plot for the differences between the two Extraction Control (EC) replicates performed in each independent experiment for Lot1 (**left**) and Lot 2 (**right**). Green line represents the mean of the results. Red lines represent the 95% confidence interval of the results. Black dots represent outliers.

Figure 2 and Figure 3 shows the variability of the results of the two types of controls used, the Sample Process Control (SPC) and the Extraction Control (EC). The Bland–Altman plots indicate that the increases between replicates in both types of controls (SPC and EC) were distributed around the mean with no discernible patterns and within the 95% confidence intervals limits (Figure 2 and Figure 3). Only one and two samples for SPC and EC, respectively, were out of the 95% confidence intervals limits; which represents 3.57% 7.14% for the Lot1 and 2.94% and 5.88% for the Lot2. Consequently, the variability was low in both cases and no bias were observed, which demonstrates the robustness and low inter-assay variability of the methodology performed. The method performance, in terms of precision and recovery efficiency, is in line with the criteria outlined in ISO 15216-1:2017. The CV values for the extraction control (EC) was consistently below 3.5%, and average recovery rates for the Sample Process Control (SPC) ranged around 28.73%, which are within the expected range for wastewater matrices based on the literature using ISO-based protocols.

**Figure 4:**
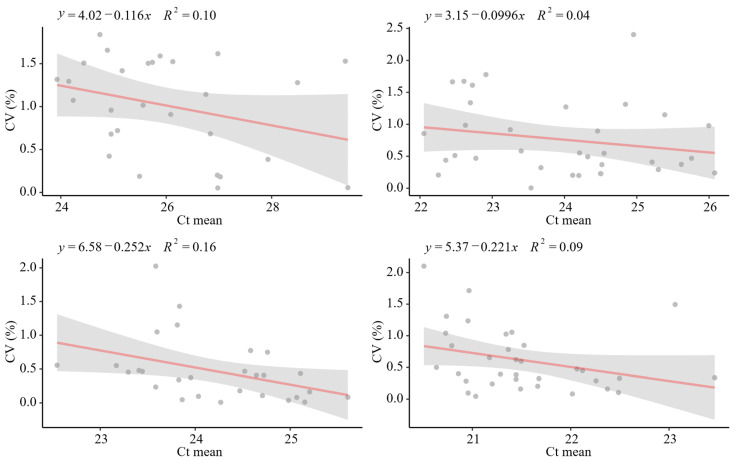
Scatter plots and linear regression for coefficients of variants for each experiment and the Ct mean obtained for SPC (**up**) and EC (**bottom**) for each batch, Lot 1 (**left**) Lot 2 (**right**).

Significantly, the coefficient of variance (CV) of the Ct values for MgV_0_ was below 2%, demonstrating the good reproducibility for both, the complete method and the extraction and quantifications step (Figure 4), in accordance with general considerations [33]. In addition, no significant correlation (*p*-value > 0.05) was found between CV and the mean of the Ct values for MgV0, except for the EC in Lot 1 which is statistically significant but just the 16% of the differences (R^2^ = 0.16) were explained by this fact (Figure 4). In the same way, no significant relation was detected between the CV and the experiment number (*p* value > 0.05), i.e., no fluctuations in the experiment along the time (Figure 4).

**Figure 5:**
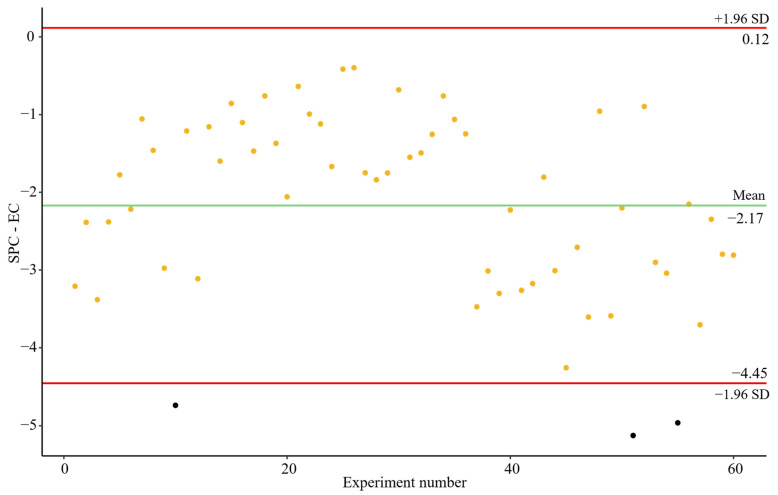
Bland–Altman plot for the differences between SPC and EC for each independent experiment. Green line represents the mean of the results. Red lines represent the 95% confidence interval of the results. Black dots represent outliers.

The increments between SPC and EC, i.e., the effect of the concentration step, were distributed around the mean without any differentiated pattern and within the 95% confidence intervals limits, apart from three points which represent 5.00% of the total experiments performed (Figure 5). The mean of the Cts differences was −2.17, indicating that, on average, 0.65 logarithmic units were lost during the viral concentration step. This calculation is based on the fact that an increase of 3.33 Cts corresponds to a loss of 1 logarithmic unit (Applied Biosystems. Thermo Fisher Scientific, 2014).

**Figure 6:**
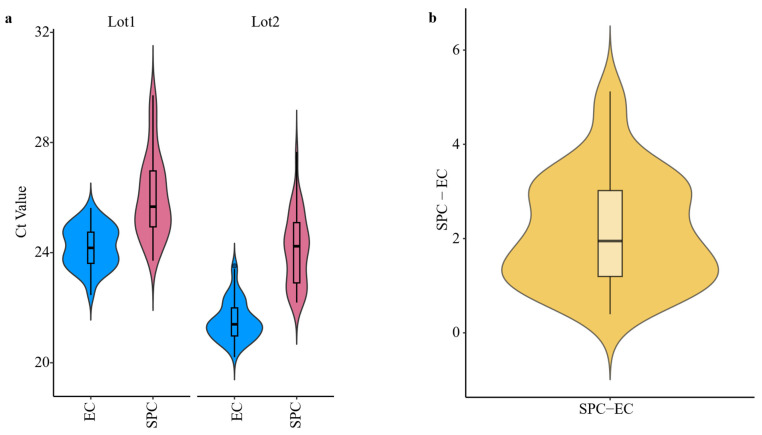
Violin plots of data distribution. (**a**) Violin plot for extraction and RT-qPCR Ct values obtained (EC) and the complete process (SPC) for both Lots analyzed (Lot 1 and Lot 2). (**b**) Violin plot of the Ct increments between SPC and EC, i.e., Ct lost during viral concentration procedure.

Table 1 shows the descriptive statistics for results obtained for the controls (EC and SPC) and increments between those controls, and Figure 6 represents the distribution of the data to assess the variability in the results generated in each step of the analytical process. The descriptive statistics presented in Table 1 and the data illustrated in Figure 6 indicate that the lowest CV was associated with the extraction control. These findings suggest that the nucleic acid extraction and amplification steps, performed using RT-qPCR, exhibited the greatest stability and the least variability throughout the process. Furthermore, the consistently low CV (~5%) observed for the entire workflow—including viral concentration, extraction, and RT-qPCR—demonstrates the overall robustness of the methodology.

Additionally, the variability between the two analyzed lots was found to be similar for both process controls. The primary distinction between them was observed in their mean and median Ct values. This outcome was expected, as Lot 1 contained a lower viral titer than Lot 2, leading to higher Ct values. Despite these differences, the methodological consistency across lots reinforces the reliability of the approach.

However, the highest degree of variability was attributed to the viral concentration step, which accounted for 53.73% of the overall variability. Interestingly, no significant association was found between viral loss and either processing time or seasonality, as indicated by the Spearman correlation test (for processing time) and the ANOVA test (for seasonal group differences). These findings suggest that other, yet unidentified, variables may play a role in influencing viral recovery during concentration, warranting further investigation to enhance the reliability and standardization of the methodology.

### 3.2. Effect of the Physicochemical Parameters on the Recovery Rates

The overall recovery rate of the 600 samples analyzed was 28.73%, where 3.50% of the samples reached recoveries higher than 100%. Most of them had some observations related to rainfalls in both previous or the sampling day, presence of foam in the sample, or an intense smell of hydrocarbons. Consequently, those samples were excluded for further analysis, assuming their composition was altered and may influence the results.

**Figure 7:**
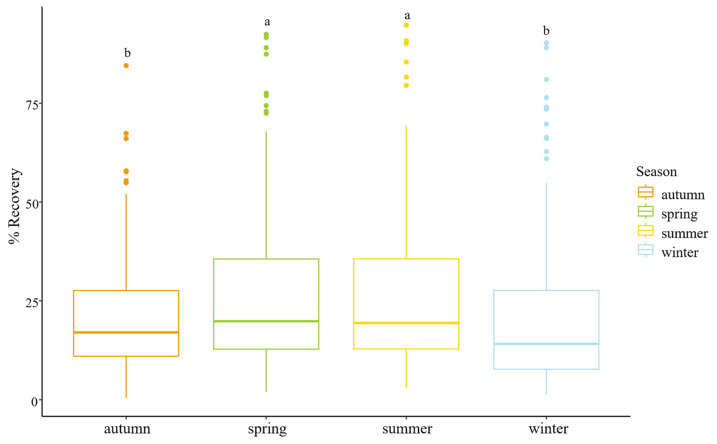
Boxplot for recovery rates grouped by climate season. Letter a above the boxes indicate that spring and summer were not significantly different from each other, while letter b indicates that autumn and winter are significantly different from spring and summer.

Samples with recoveries below 100% (*n* = 579) were further studied to know the potential effects of the relevant physicochemical factors in the recovery rates (Supplementary Material, Figure S1). Recovery efficiency showed no significant relationship with physicochemical parameters such as pH and conductivity (*p* > 0.05). However, a statistically significant effect of seasonality was observed (Kruskal–Wallis test, *p* < 0.05). Specifically, spring and summer samples showed higher recovery rates than autumn and winter (Figure 7). This suggests that climatic conditions such as temperature or humidity may affect viral concentration efficiency, possibly by altering sample composition or flocculation dynamics.

The samples used in this study came from 12 different areas. Given the inherent heterogeneity of wastewater, even within the same sampling site, investigating potential correlations between viral concentration efficiency and sample origin was of particular interest. To assess these relationships, statistical assumptions of normality and homogeneity of variances were tested using the Shapiro–Wilk (*p*-value > 0.05) and Levene tests (*p*-value > 0.05), respectively, confirming that the data did not follow a normal distribution and exhibited heterogeneous variances. Consequently, a non-parametric Kruskal–Wallis test was performed, revealing that at least one sample exhibited significantly different recovery rates compared to the others. These findings suggest that certain site-specific factors may influence viral recovery, highlighting the need for further investigation into the role of sample composition and environmental conditions in wastewater-based epidemiology. Differences in viral recovery were also observed across sampling sites. A post hoc Dunn test, with a Benjamin–Hochberg correction method, showed that samples 4, 10, and 11 were statistically different from samples 1, 3, 6, and 12, also sample 9 related to sample 10 (*p*-value > 0.05) (Table 2 and Supplementary Material Figure S2).

This suggests that local factors, such as industrial discharges or shared infrastructure, could affect wastewater composition and thus viral recovery.

In summary, while the methodology demonstrated high overall robustness, variability in viral recovery was influenced by environmental seasonality and sampling location, but not by pH or conductivity. These findings underscore the importance of contextual controls and metadata in interpreting WBE results and optimizing protocols.

## 4. Discussion

Wastewater samples are very heterogenous, and the presence of inhibitors should be investigated to be able to obtain virus and RNA recovery information. In order to achieve this issue, and according to [26], we have assessed the reproducibility of the Aluminum-based adsorption–precipitation method for virus detection in wastewater samples by the inclusion of two process controls: a Sample Process Control (SPC) and an Extraction Control (EC) [12].

Aluminum-based adsorption–precipitation method is usually employed to obtain viral concentration in wastewater analysis. Several studies comparing different viral concentration methods have been already performed, especially since COVID-19 pandemic, where WBE attracted a lot of interest [34,35,36]. However, to our knowledge, there are not studies available assessing its robustness, the variability of each phase, and the potential effect of relevant physicochemical factors using a high sample size (*n* = 612). In this sense, high variability in recovery rates has also been reported in aluminum-based adsorption–precipitation method [17,37], what corroborates the importance of using process controls to estimate the original virus load in each sample.

In this study, the analysis of the process controls demonstrated that the overall methodology exhibits a satisfactory reproducibility and robustness; low variability was observed, as the data were generally distributed around the mean difference in the Bland–Altman plots, with no significant dispersion. However, the concentration step exhibited the highest heterogeneity (CV > 50%), making it the phase that most significantly impacts the final results.

Based on the quality metrics evaluated (CV, recovery efficiency, and variability), our method demonstrates satisfactory robustness and reproducibility. Specifically, a CV below 5% for RT-qPCR-based quantification indicates good analytical precision, consistent with MIQE guideline thresholds. The viral recovery performance, with a mean of ~28%, is similar or superior to that reported in recent inter-method comparison studies, including those by [38,39]. These findings reinforce the method’s applicability for routine wastewater-based epidemiology (WBE), where process variability and matrix effects are expected. On average, MgV was recovered at ranges of 28.73 ± 1.40% (Mean ± Standard Error), where some samples presented rates over 100%, which were excluded for further analysis because observations about them were collected at sampling time. Furthermore, this fact has been previously reported by [40], which could occur due to the heterogeneity of the wastewater samples composition, for example, for the presence of humic acid and proteins which may enhance the flocculation phase [41]. Excluding those data, recovery rates ranged around 23.68 ± 0.78% (M ± SE). Interestingly, these recoveries were higher than those reported in the literature [25,35,37,40]. Several factors may influence the viral recovery rates. These include variables related to the concentration process itself, such as the buffer elution used, flocculation time, or the type of flocculant employed [24], as well as the wastewater characteristics, such as pH [24,42]. Our results indicate some temporal variation in viral loss (Figure 5). However, no statistically significant correlation was found between the increase in Ct values between SPC and EC and the experiment number when analyzed in chronological order.

The correlation between recovery rates and the physicochemical parameters was also studied and significant correlations have been found for season and samples. The first one could be explained by different temperatures and humidity during the different climate seasons. Moreover, climate could also influence the composition of the samples that could affect the extraction and concentration process. The correlation between samples and recovery rates may be due to the influence of the sample composition, particularly regarding the organic and inorganic molecules that can affect conductivity and impact the methodology [41,43]. Specifically, in this study, each sample originates from the sewer network at different locations within a city. As a result, some characteristics may be shared between samples depending on the area they come from, influencing their composition. This is the case for samples 10 and 11, which are significantly different from others, which are closely related due to several factors: their geographical proximity, the fact that the flow of sample 11 is part of the flow of sample 10, and because they monitor an identical number of inhabitants. It is therefore reasonable that the composition and, consequently, the results of these two samples differ significantly from the others. Additionally, another factor that could contribute to these differences is the discharge of specific compounds by businesses located in different areas, which may also affect sample composition. This could potentially explain the differentiation of sample 4 from several of the remain analyzed samples.

Since a relationship between the efficiency of the methodology and both the origin of the sample and the season has been observed, likely due to their influence in sample composition, future studies exploring the potential relationship with other parameters could be of interest. These may include chemical oxygen demand (COD) to assess the organic matter present in the samples, as well as the concentration of other compounds such as phosphorus, nitrogen, or additional indicators of fecal contamination e.g., Pepper Mild Mottle Virus (PMMoV), crAssphage, or somatic coliphages, as other authors have previously used for normalization [44,45,46]. Such investigations could help evaluate the correlation of these concrete parameters with the effectiveness of the methodology and ultimately contribute to the development of a model that incorporates these variables for standardizing the method.

Comparing results across different studies and methodologies is challenging, as each employed varying sample volumes and distinct viral surrogates as controls. However, the AlCl₃ based adsorption–precipitation method used in this study achieved recovery rates comparable to those obtained with other electronegative membrane adsorption–precipitation methods involving prior acidification (26.7 ± 15.3%), as well as to ultracentrifugation using Centricon Plus-70 filters (28 ± 9.1%), as reported by [38]. Different studies [39,47], suggest that ultracentrifugation is generally the most effective concentration method. The first of these studies identified ultracentrifugation as the most efficient technique, with a recovery rate of 25 ± 6%, which is slightly lower than the rate observed in our study, followed by AlCl_3_-based methods. The second study concluded that this methodology is followed by polyethylene glycol (PEG) precipitation, skimmed milk flocculation (SMF), and electronegative membrane filtration, ordered by effectiveness.

Nonetheless, when selecting a concentration method, various practical considerations must be taken into account, such as cost, time consumption, and equipment requirements. Ultracentrifugation is the most expensive option, while PEG precipitation and SMF are time-intensive. Given these constraints, aluminum-based adsorption–precipitation emerges as a strong alternative because of its cost-effectiveness, rapidity, robustness (demonstrated in this study), and it does not require specialized equipment. Moreover, as mentioned before, it could also reach similar recoveries as ultracentrifugation. Therefore, by carefully controlling and understanding the parameters that affect recovery efficiency—as explored in this and future studies—aluminum-based adsorption–precipitation could serve as a viable candidate for a standardized viral concentration method.

## 5. Conclusions

Despite the overall robustness of the aluminum-based adsorption–precipitation method, the results of this study indicate that the viral concentration step significantly contributes to the variability of the outcomes. These findings highlight the importance of incorporating multiple process controls to evaluate the performance of the methodology, particularly in wastewater-based epidemiology (WBE), where sample characteristics can be highly variable. This approach also enables more accurate extrapolation of the original viral load in each sample, leading to more reliable results.

Although no significant correlation was observed between recovery rates and parameters such as pH or conductivity, environmental factors (e.g., season) and specific sample characteristics were found to influence the efficiency of the methodology.

Overall, the aluminum-based adsorption–precipitation method shows strong potential as a standardized approach for viral concentration in WBE due to its notable advantages. Further research should explore additional parameters—such as organic matter content or other interfering compounds—that may affect the method’s effectiveness, in order to enhance understanding, improve control, and support standardization efforts.

## Figures and Tables

**Table 1:** Descriptive statistics for results obtained for the controls (EC and SPC) and increments between those controls.

Control	Lot	Mean	Median	Sd	Min	Max	Range	CV (%)
EC	Lot 1	24.21	24.17	0.75	22.46	25.62	3.17	3.09
	Lot 2	21.51	21.40	0.70	20.20	23.52	3.32	3.28
SPC	Lot 1	26.03	25.67	1.49	23.71	29.72	6.01	5.74
	Lot 2	24.11	24.23	1.30	22.19	27.66	5.48	5.39
SPC-EC		2.17	1.95	1.17	0.40	5.12	4.73	53.75

**Table 2:** Statistically significant results (*p*-adjusted < 0.05) for the recovery rates comparison between sample obtained in post hoc Dunn test.

Comparisons		*p*-Adjusted
Sample_1	Sample_4	0.023
	Sample_10	0.020
	Sample_11	0.019
Sample_3	Sample_4	0.021
Sample_4	Sample_6	0.017
Sample_10	Sample_3	0.012
	Sample_6	0.008
	Sample_9	0.033
	Sample_12	0.015
Sample_11	Sample_3	0.016
	Sample_6	0.015
	Sample_12	0.028
Sample_12	Sample_4	0.028

## Data Availability

Data are available upon request to the corresponding author.

## Supplementary Materials

The following supporting information can be downloaded at: https://www.mdpi.com/article/10.3390/microorganisms13061186/s1; Figure S1: Scatter plots of recovery rates against sample’s pH, Conductivity, Temperature, and sample itself; Figure S2: Boxplot for recovery rates grouped by sample; Table S1: Recovery_f_q_factors.
