# Supplementary material for: Assess the Variability and Robustness of an Aluminum-Based Adsorption–Precipitation Method for Virus Detection in Wastewater Samples
Source: Microorganisms. 2025 May 23;13(6):1186. doi: 10.3390/microorganisms13061186 (PMC12195461; doi:10.3390/microorganisms13061186)

Supplementary material

Figure S1: Scatter plots of recovery rates against sample's pH, Conductivity, Temperature, and sample itself.

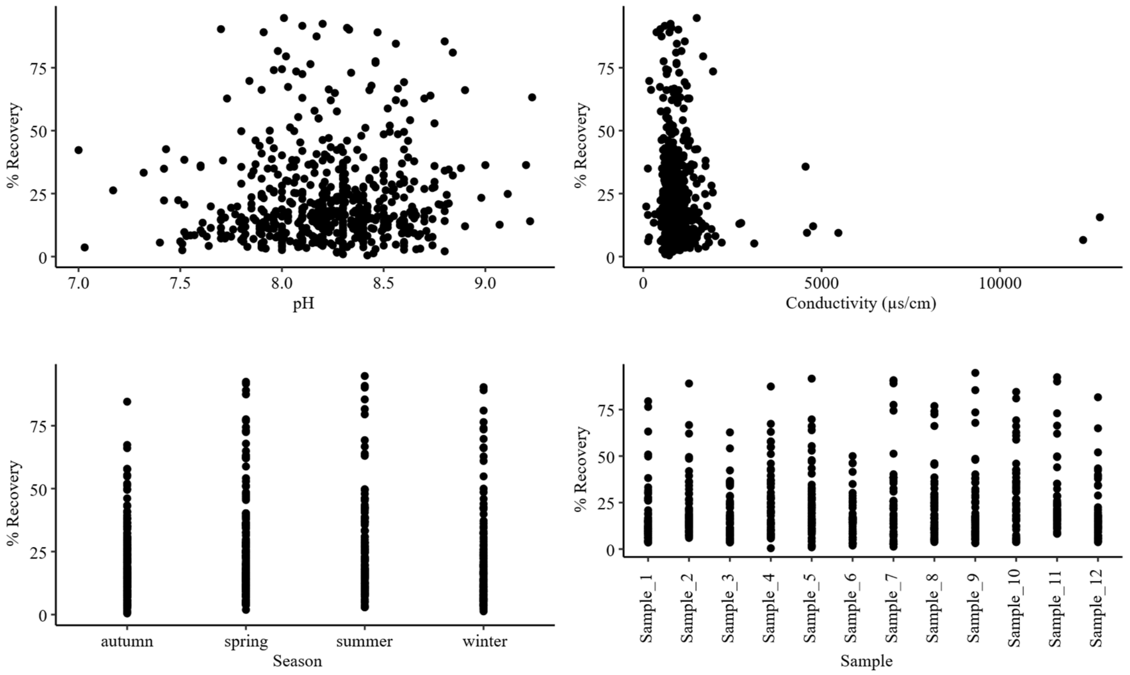

Figure S2: Boxplot for recovery rates grouped by sample.

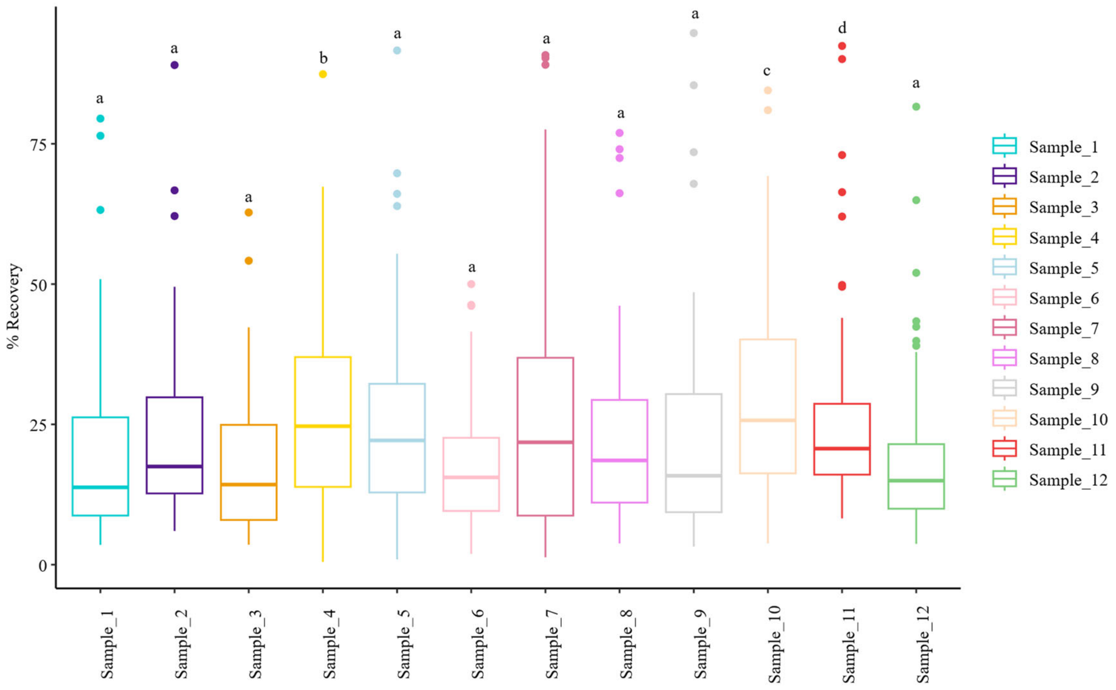

Supplement: Supplementary file 1 [file microorganisms-13-01186-s001.zip › Supplementary_material_mengovirus_paper.pdf]
